# Challenges in interpreting Mendelian randomization studies with a disease as the exposure: Using COVID-19 liability studies as an exemplar

**DOI:** 10.1038/s41431-025-01840-x

**Published:** 2025-03-31

**Authors:** Siyu Chen, Ying Liang, Jacky Man Yuen Mo, Queenie Ho Yi Li, Baoting He, Shan Luo, Stephen Burgess, Shiu Lun Au Yeung

**Affiliations:** 1 School of Public Health, LKS Faculty of Medicine, the University of Hong Kong, Hong Kong Special Administrative Region, China; 2 MRC Biostatistics Unit, Cambridge Institute of Public Health, University of Cambridge, Cambridge, United Kingdom

**Keywords:** genetic liability, COVID-19, binary exposure, dichotomous exposure, Mendelian randomization

## Abstract

Mendelian randomization (MR) studies using disease as exposures are increasingly prevalent although any observed associations do not necessarily imply effect of diseases. To illustrate this challenge, we conducted a systematic review of MR studies focusing on COVID-19 consequence. We hypothesized if outcome genome-wide association studies (GWAS) were conducted before COVID-19 pandemic in late 2019, any observed associations in these studies were unlikely to be driven by COVID-19. We systematically searched PubMed, EMBASE, and MEDLINE for all MR studies published between 1 January 2019 and 20 May 2023. Inclusion criteria included MR studies which used COVID-19 as the exposure and designed to assess COVID-19’s impact on health outcomes. We extracted relevant information, such as result interpretation and relevance assumption assessment. This review was registered at PROSPERO (CRD42023421079). Amongst 57 included studies, 45 studies used outcome GWAS published prior to 2019 whilst the remaining studies likely used outcome GWAS containing data collected before 2019. Relevance assumption was assessed mainly by p values. A total of 35 studies showed an association of COVID-19 liability with health outcomes. Regardless of the results, 45 studies attributed these as evidence (or lack of evidence) of COVID-19 consequence. In MR studies using disease liability as exposure, relevance assumption should consider the prevalence of the disease in the outcome GWAS in the context of 2 sample Mendelian randomization study rather than p values/ F-statistic alone. Even when these are verified, these studies likely suffered from pleiotropy, making corresponding interpretation as effect of disease challenging.

## Introduction

Mendelian randomization studies are increasingly used to address important public health questions given it is more resistant to confounding with the use of genetic variants randomly allocated at conception that is analogous to randomization in randomized controlled trials.^1^ As the number of genome-wide association studies (GWAS) of diseases grows, providing a larger pool of candidate instruments (e.g. from 65 single nucleotide polymorphisms (SNPs) in 2012 to 611 SNPs for type 2 diabetes in 2024),^2, 3^ recent studies have attempted to use Mendelian randomization to assess the impact of diseases on other diseases and traits,^4–6^ as well as to assess possible reverse causation in the context of bidirectional Mendelian randomization design.^7, 8^

Interpreting results from Mendelian randomization using instruments of diseases can be challenging. In these studies, although the absence of association may support the lack of evidence for the disease’s effect on outcomes, the presence of associations observed has sometimes been interpreted as having an effect of the disease on the outcomes of interest, which is technically incorrect given the exposure is liability of disease instead of diagnosis.^9–11^ Correspondingly, the presence of associations in these studies can have various interpretations, depending on our understanding of the subject matter and the characteristics of the underlying population.^12, 13^ For example, if a study of genetic liability of type 2 diabetes is conducted amongst populations where type 2 diabetes was absent, any associations observed are unlikely to be the consequences of type 2 diabetes, as this directly violates the relevance assumption (i.e. since variants would not be related to type 2 diabetes in a population where type 2 diabetes was absent) in the Mendelian randomization design given the participants have their exposure status fixed (i.e. no type 2 diabetes).^14^ Conversely, such studies conducted amongst adult populations where type 2 diabetes is present in the population are more difficult to interpret, given any observed associations represent the effects of underlying continuous risk factors related to type 2 diabetes (e.g. glucose, glycated hemoglobin (HbA1c), and body mass index) and the effect of type 2 diabetes itself.^15^ Whilst these issues have been touched upon in previous papers,^9^ few papers attempted to explore these issues using empirical data, likely because approaches such as stratifying upon disease status when assessing genetic risk score of diseases with outcomes will inevitably introduce collider bias,^16^ where disease status is a common effect of genetics and confounders of disease-outcome associations, rendering the observed associations uninterpretable.

To demonstrate the complication of interpreting Mendelian randomization studies with disease as exposure whilst reducing the likelihood of collider bias, we took advantage of the COVID-19 pandemic given participants in datasets collected prior to the COVID-19 pandemic (i.e. late 2019) would have fixed exposure status regarding COVID-19. Therefore, any of the associations observed in such studies using pre-pandemic studies would not be an impact of COVID-19. To this end, we conducted a systematic review of all Mendelian randomization studies which 1) used COVID-19 as the exposure and 2) intended to explore the effect of COVID-19 on disease outcomes (i.e. long COVID-19) as the stated objectives. We hypothesized that if the outcome GWAS used in these studies were conducted before the COVID-19 pandemic in late 2019, these studies could still have associations although these could be reflective of properties other than the consequence of COVID-19 and hence interpretation of any associations as evidence of long COVID-19 are likely incorrect.

## Methods

We conducted this systematic review with reference of the Preferred Reporting Item for Systematic Reviews and Meta-Analysis (PRISMA) statement (See Supplemental material),^20^ where the corresponding protocol was registered at the International Prospective Register of Systematic Review (PROSPERO) on 4 July 2023 (CRD42023421079).

### Source of information and study selection

We included Mendelian randomization studies that evaluated the associations of genetic liability to COVID-19 as exposures with health outcomes. COVID-19 exposures included severity (A), hospitalization (B), and susceptibility (C). Two reviewers independently searched studies published in PubMed, EMBASE, and MEDLINE from 1 January 2019 to 20 May 2023. We used the search strategy with different combinations of search items: (‘covid-19’ OR ‘sars-cov-2’ OR ‘2019-nCoV’ OR ‘novel coronavirus’ OR ‘severe acute respiratory syndrome coronavirus 2’ OR ‘COVID’) AND (‘mendelian randomization’ OR ‘genome-wide association study’ OR ‘mendelian randomisation’ OR ‘GWAS’). We selected human studies published in English using PubMed, EMBASE, and MEDLINE filters. We also manually checked the reference lists of the included studies to identify additional studies. Detailed search strategies for each database can be found in the Supplemental material.

### Eligibility criteria

We excluded studies that (i) were duplicated among the databases; (ii) were not research articles (e.g. reviews, commentaries, corrections) or without sufficient original data for critical appraisal (e.g. conference proceedings); (iii) were not Mendelian randomization studies; or (iv) did not include genetic liability to COVID-19 or COVID-19 phenotypes as the exposures.

### Data extraction

For each included Mendelian randomization study, the two reviewers extracted key information using a modified template based on the PRISMA checklist^21^ and the guidelines for strengthening the reporting of observational studies in epidemiology using Mendelian randomization (STROBE-MR).^22^ Two reviewers independently screened the titles and abstracts of all the studies searched, and full text if necessary. Extracted information included the name of the first author, year of publication, type of Mendelian randomization study (1 sample Mendelian randomization, where instrumental variables (IV)-exposure and IV-outcome associations were estimated from the same data; or 2 sample Mendelian randomization, where IV-exposure and IV-outcome associations were estimated from different data), details on the exposure and outcome GWAS, information related to relevance assumption (e.g. selection criteria of instruments and evaluation of its strengths), findings of the studies and its interpretation, whether the studies used STROBE-MR checklist, and whether the studies were published before STROBE-MR published on 26 October 2021. Given this study evaluated whether COVID-19 was present in the outcome GWAS used in previous Mendelian randomization studies, we also considered the year when the outcome GWAS was published or the time of data collection of the outcome GWAS published after 2019 (See Supplemental Table 1).

### Main reporting outcome

As the objective of this review was to demonstrate whether studies using genetic liability of COVID-19 can generate implausible findings (e.g. presence of association in populations with no COVID-19 cases), the main outcome of this review was to assess whether the included studies were statistically significant.

### Risk of biases in each study

There is no formal checklist for bias assessment for Mendelian randomization studies whilst not all items in the STROBE-MR checklist are relevant to the quality of the studies.^22^ Given the objective of this study was to assess the validity of instruments related to COVID-19 liability rather than conventional reviews of Mendelian randomization studies,^23^ we focused primarily on the authors’ assessment of the relevance assumption.

Given this is a systematic review summarizing findings from published studies, ethical approval was not required.

## Results

**Figure 1:**
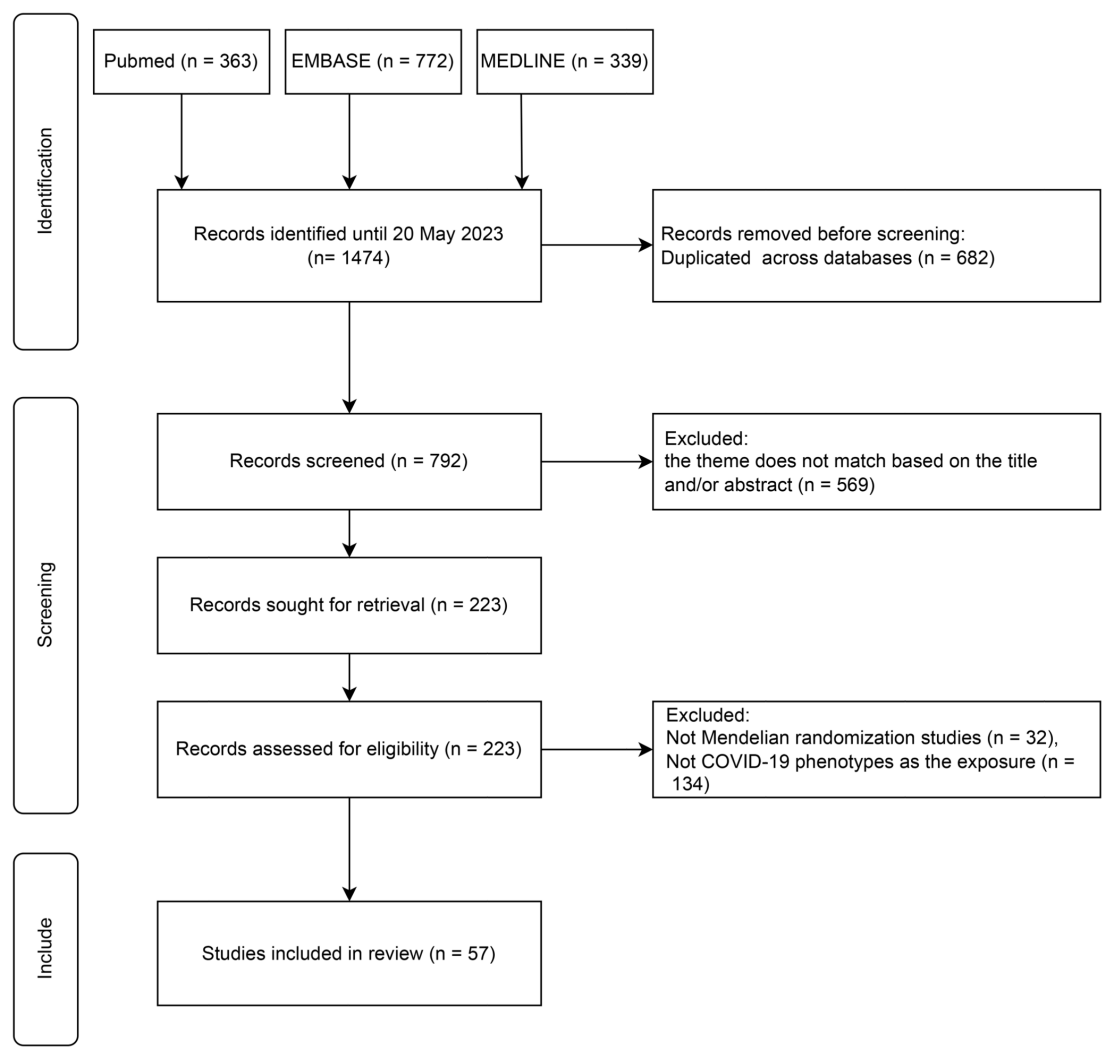
Flow chart of this study.

The initial search identified 1,474 studies, including 363 from PubMed, 772 from EMBASE, and 339 from MEDLINE. After removing duplicate studies, this gave 792 studies. An additional 569 studies were excluded based on screening of the title and/or abstract. Amongst the remaining 223 studies, 166 studies were further excluded either they were not Mendelian randomization studies, or did not consider COVID-19 as an exposure, or were duplicate studies. In total, 57 eligible Mendelian randomization studies were included in this review (Figure 1), where these studies were published between March 2021 and May 2023 (See Supplemental Table 1).

**Figure 2:**
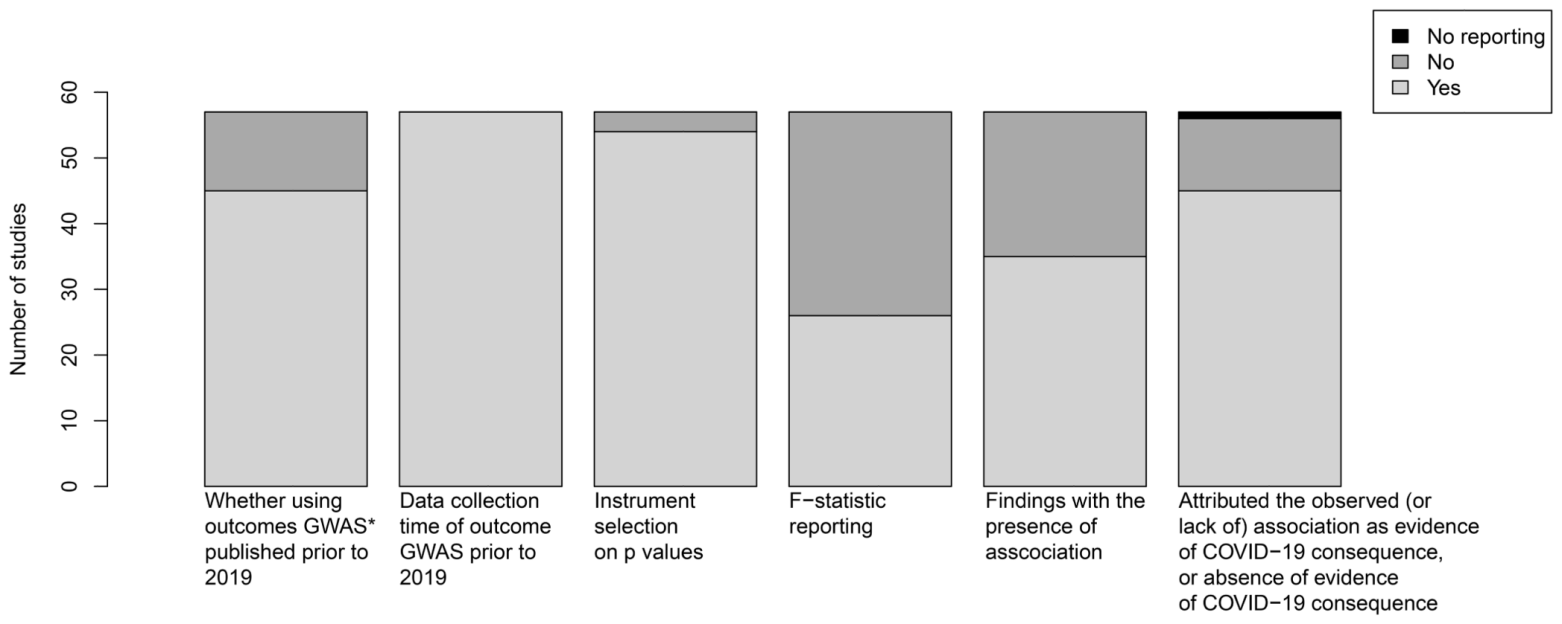
Summary of the results included in this review. GWAS*: genome-wide association studies.

The characteristics of the included Mendelian randomization studies were summarized in Figure 2. All studies were 2 sample Mendelian randomization studies. The majority of these studies (52) used genetic instruments identified from the COVID-19 Host Genetics Initiative^24^ whilst other studies used instruments derived from smaller GWAS amongst Italian and Spanish population^25^ or the study of Genetics of Mortality In Critical Care (GenOMICC).^26^ There was a comprehensive range of health outcomes, ranging from cardiovascular diseases,^27^ mental health,^17^ to cancers.^28^ Amongst these studies, 45 used outcome GWAS published prior to 2019. Although the remaining 12 studies used outcomes published after 2019, these outcome GWAS likely included data collected prior to 2019. For example, the study by Jia et al.,^27^ used the GWAS of FinnGen published in 2019 although the datasets used were collected from 2017 and were released in quarter 4 of 2019. In terms of assessment of the relevance assumption, 54 out of 57 studies selected instruments based on p values whilst 26 studies also reported F-statistic. For example, Luykx et al.,^17^ reported “We first selected all relevant single-nucleotide variants identified in each GWASs as having reached a selection p value threshold < 5 × 10^−8^” and “we estimated the F-statistic from first-stage regression to evaluate instrument strength” (Page 5).

The outcomes were categorized as cardiovascular-related (10 studies), metabolic-related (5 studies), cancers (3 studies), respiratory related (1 study), longevity (4 studies), mental or neurological (14 studies), immunological (6 studies), blood traits (4 studies), reproductive (3 studies), cytokines (3 studies), dermatological (1 study), hormones (1 study), multiple phenotypes (1 study), and muscle-related (1 study). Amongst the 57 studies, 35 (61%) studies showed a possible association between genetic liability to COVID-19 with health outcomes despite participants in the outcome GWAS used in all these Mendelian randomization studies unlikely had COVID-19 based on the year of data collection and hence was unlikely driven by COVID-19. However, regardless of whether the associations were significant, 45 (79%) of these studies attributed the observed (or lack of) association as evidence of COVID-19 consequences, or the lack of association considered as absence of evidence for a COVID-19 consequence. For example, Liu et al.,^18^ reported “There was no evidence derived from this work to support causal relationships between COVID-19 susceptibility/severity and non-alcoholic fatty liver disease” (Page 742). Among the remaining 12 studies which did not attribute the observed (or lack of) association as evidence of COVID-19 consequences, 6 studies yielded null results while the other 6 studies showed associations. A total of 11 out of 12 has statement or description of the results. Among these 11 studies, 10 studies used the phrase “genetic liability/genetic susceptibility/genetic predisposition/liability of COVID-19”, for example, Ran et al.,^19^ reported “genetic predisposition to COVID-19 was not causally associated with the risk of systemic lupus erythematosus” (Page 1), while Gao et al.,^29^ reported “We found that as COVID-19 genetically increased, the risk of endometrial cancer had an increased trend” (Page e85). Considering that STROBE-MR checklist was published on 26 October 2021, only one out of 51 studies published after this date included the relevant checklist.

## Discussion

In this systematic review, we found the majority of Mendelian randomization studies using liability to COVID-19 as an exposure included outcome GWAS conducted prior to the COVID-19 pandemic. Despite the absence of COVID-19 in these outcome GWAS, several studies reported potential associations of genetic liability to COVID-19 with disease outcomes as the consequence of COVID-19 on disease outcomes including coronary artery disease,^30^ Alzheimer’s disease,^31^ and hypertensive disorders.^32^ Unfortunately, these studies attributed the observed association as a consequence of COVID-19,^30–34^ which is biologically implausible. Our study provides empirical evidence highlighting the complexities and potential pitfalls of Mendelian randomization studies using disease status as an exposure, as association can still be observed using outcome data sources with invariant exposure status (i.e., absence of COVID-19). These findings have substantial implications for the interpretation of related studies, such as bi-directional Mendelian randomization design often used to assess reverse causation, or Mendelian randomization studies aiming to assess the impact of one disease on other diseases. Based on this review, we reported two main issues: the meaning of relevance assumption, the challenges of Mendelian randomization studies using disease as exposures. We also proposed suggestions for improved design, and a proper interpretation of corresponding Mendelian randomization studies.

### Rethinking the “relevance” assumption in the context of 2 sample Mendelian randomization for diseases as exposures – Is the disease present in the outcome GWAS?

The relevance assumption implies the genetic instrument is strongly associated with the phenotype of interest, and is the only assumption that can be tested empirically. For example, previous 1 sample Mendelian randomization studies assessed the association of genetic instruments (e.g. aldehyde dehydrogenase 2 (*ALDH2*)) in exposures (alcohol) using regression analyses in a sample, and also used the same sample to derive the causal estimate of exposures in outcome (cognitive function).^35^ However, in the context of 2 sample Mendelian randomization studies, this approach may not be possible, either because the exposure is not present or the data is not accessible by the researchers. Therefore, researchers often rely on F-statistic or p values reported in the exposure GWAS (1^st^ sample) for assessment of relevance assumption. To ensure the associations identified in the 1^st^ sample (exposure GWAS) can be applied to the 2^nd^ sample (outcome GWAS), which cannot be assessed, it is often conventional to use strong instruments (i.e., low p value with the phenotype in the 1^st^ sample to guard against false positives) to ensure the instruments can be assumed to be associated with the phenotype in the 2^nd^ sample. Similarly, it is also common to use the GWAS of exposures and outcomes from the same ethnic populations as the variant-phenotype association could differ by ethnicities, i.e. a variant may strongly predict a phenotype in Europeans but not in East Asians.^36^ However, this review highlighted that in the case of Mendelian randomization studies concerning diseases as an exposure, such as COVID-19, researchers may not have considered whether the exposure varies in the 2^nd^ sample. This review showed that the majority of Mendelian randomization studies have only relied on statistics for assessment of relevance assumption without evaluating the epidemiologic nature of the data. For example, if there are no COVID-19 cases in the 2^nd^ sample, then by definition, the relevance assumption would be violated even if the instruments for COVID-19 are very strong statistically, rendering the results in the corresponding Mendelian randomization studies uninterpretable.

### Complications for Mendelian randomization studies using disease traits as exposures – How prevalent is the disease in the outcome GWAS?

**Figure 3:**
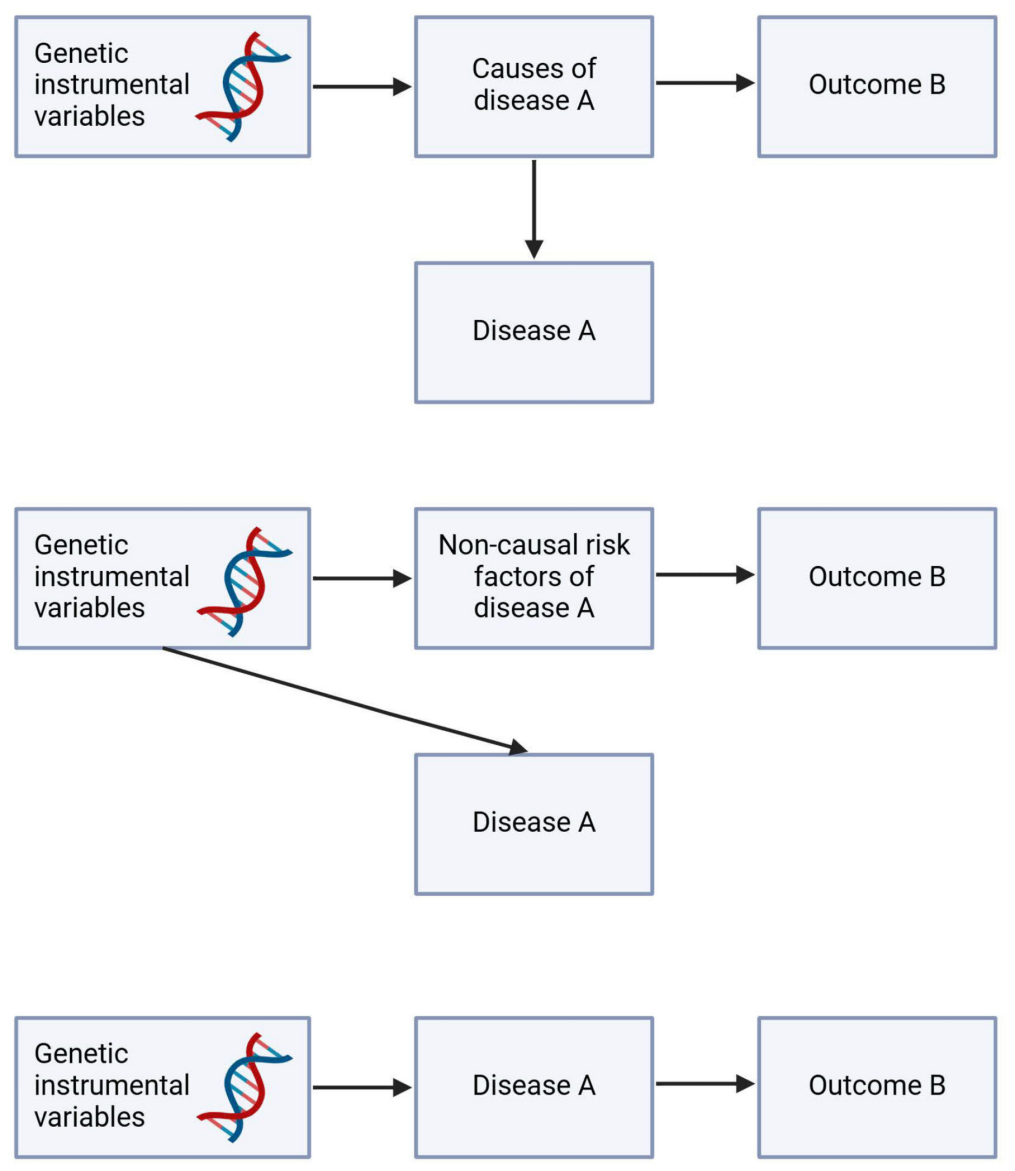
Associations with genetic liability to disease can be reflective of causes, risk factors, and consequences of the diseases by directed acyclic graph (DAG).

**Figure 4:**
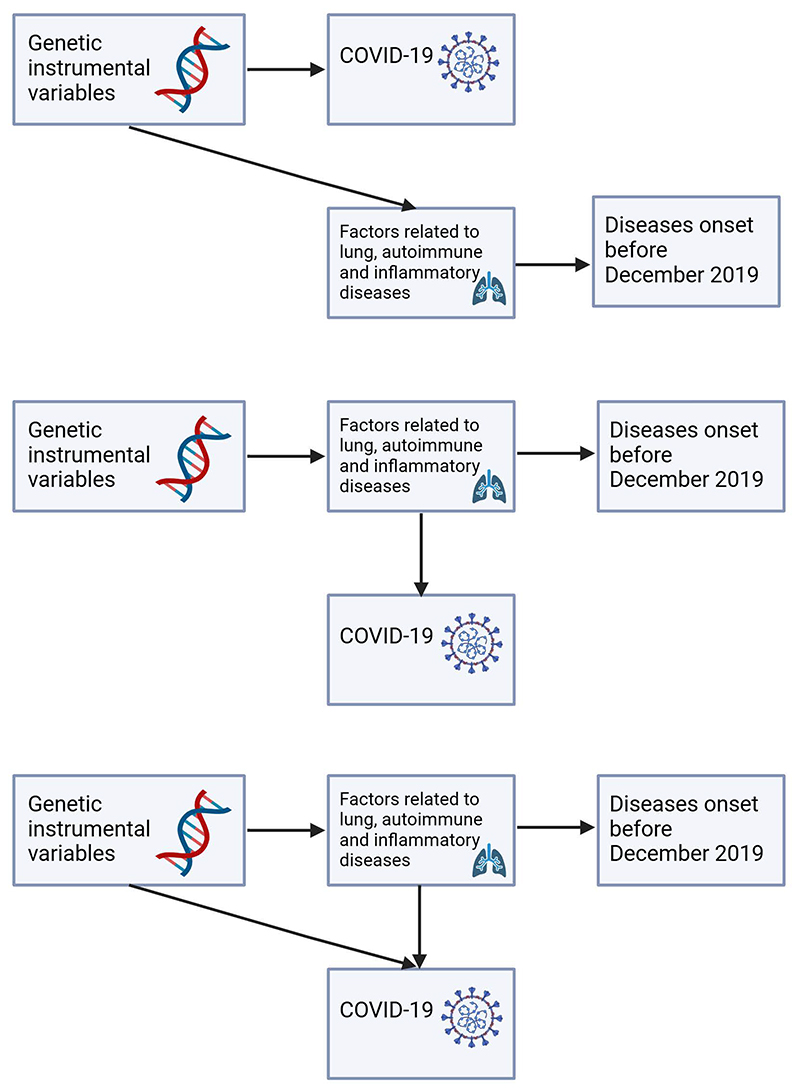
Possible scenarios leading to an association with genetic liability to diseases in the context of COVID-19 Mendelian randomization studies.

One of the motivating reasons for conducting this review is to assess whether there will be any associations between COVID-19 instruments with disease outcomes where COVID-19 is invariant. The observed associations in many of the included studies have highlighted the challenges in interpreting Mendelian randomization studies with binary disease traits.^9, 12^ Associations with genetic liability to disease can be reflective of causes, risk factors, and consequences of the diseases (See Figure 3).^12^ For example, the potential association of COVID-19 liability to type 2 diabetes may simply be a risk factor signal related to glycemic traits although earlier Mendelian randomization studies did not indicate a causal role of type 2 diabetes liability and glycemic traits in COVID-19 risk.^37, 38^ It could also be a reflection of pleiotropic effects of COVID-19 instruments, where some of the variants are related to lung, autoimmune, and inflammatory diseases (See Figure 4), which may, in turn, be related to other chronic diseases such as cardiometabolic diseases and Alzheimer’s diseases,^39, 40^ although the issue of horizontal pleiotropy can be applied to most Mendelian randomization studies. However, genetic liability to continuous trait is different from genetic liability to a dichotomous trait, which is often defined based on a continuous variable that define the trait.^9^ For continuous traits (as exposures), changes in the genetic IV will lead to, on average, changes in the continuous trait and hence imposing an effect on the outcomes due to changes of the exposure, while the corresponding binary trait can remain fixed. For example, hypertension is defined based on blood pressure thresholds. Hence, changes in the genetic instrumental variables will lead to an increase of blood pressure and impact disease outcomes although hypertension status may not necessarily change concurrently if the threshold for hypertension is not crossed. Hence, this also emphasizes the point on inferring the observed associations as a reflection of genetic liability instead of diagnosis, which unfortunately, was a main issue in many of the included studies. Nevertheless, the translational aspect of these findings of genetic liability to diseases is unclear unless we also take into consideration the overall characteristics of the population, such as the prevalence of the disease in the outcome GWAS. A higher prevalence of the exposure disease in the 2^nd^ sample may increase the likelihood that the observed association reflects the consequence of the disease rather than its causes or risk factors,^15^ and is likely an important aspect for consideration by many Mendelian randomization researchers who used diseases as exposures.

### *Extension to other Mendelian randomization using dichotomous* exposures

The same rationale described above can be applied to dichotomized exposures. For example, it would be of public health interest to evaluate long term effects of cannabis use with Mendelian randomization given the increasing trend of such use globally and there are instruments available from a relevant multiethnic GWAS.^41, 42^ However, conduct of a 2 sample Mendelian randomization study to investigate this research question will need to consider prevalence of cannabis use in the underlying outcome GWAS given variability of its use in different regions and countries.^43^ It might be appropriate to conduct such study in United States but is less likely to be valid in other places with tighter controls, such as China.

Furthermore, whether genetic liabilities to different binary traits should be evaluated separately would be worth more in-depth discussion. With regards to infectious disease, genetics itself cannot cause infectious disease risk because the only “cause” is an exogenous factor, the pathogen (e.g. influenza virus causing influenza). Therefore, genetic liability of infectious disease likely included factors which contribute to increased susceptibility to infection, such as variants related to higher body mass index, or variants which were involved in host-parasite genetic interactions (e.g., sickle cell anaemia and malaria).^44, 45^ Hence, this could have contributed to differences in genetic predictors of infectious diseases within the same population or across settings.^44^ These could have implications regarding the relevance assumption given these variations. With regards to non-communicable diseases, genetics can cause the disease as these genetic variations contribute to varying biological processes, such as elevated cholesterol, blood pressure, and anthropometric measurements.^46^ Therefore, genetic liability of non-communicable disease likely included factors that alter these biological factors. On the other hand, genetic liability to behavioural condition is partly dependent on the environment, such as availability of exposures (e.g. accessibility to alcohol), and social norms, and hence may have explained differences in genetic predictions on different populations (e.g. *ALDH2* polymorphism only predicts alcohol use in men but not women because women were not drinking at all given social norm in China).^16^ Nevertheless, regardless of the type of binary traits concerned, there are always a possibility of pleiotropy due to the underlying different causal structures that link genetics with the binary trait. It would also be important to check if the trait actually exists in the outcome GWAS as a basic check given this seems to be a most plausible approach using published GWAS.

### Examples of correct interpretation, incorrect interpretation, and inconsistent interpretation

We noticed there were substantial variations in how these associations were described and interpreted. Even amongst studies which correctly mentioned any observed associations were reflection of genetic liability to COVID-19 (e.g. “…host genetic liability to severe covid-19 was causally associated with increased mean platelet volume (MPV) and reduced platelet count”(page 4735)^47^), certain studies interpreted the findings incorrectly, such as attributing the lack of association as absence of evidence for an effect of COVID-19 in the diseases concerned (e.g. “Similarly, contrary to one study, we found no evidence of COVID-19 influencing risk of psychiatric disorders”(page 8)^17^), where some of the excerpts from these studies were listed in the textbox for illustrative purposes (Textbox 1).^47–51^

In this study, we highlighted the issues surrounding Mendelian randomization studies that consider diseases as exposure, using empirical evidence from COVID-19 Mendelian randomization studies. However, there are some limitations. First, there are substantial variations in the quality of the studies included in our review, issues such as insufficient information on details of the GWAS used, corresponding units, and misreporting of information were not uncommon (See Supplemental Table 1 comment column for details). Although this does not directly relate to the main objective of this review, which is to assess the appropriateness of the choice of outcome GWAS, it does highlight the issue of poor quality. Mendelian randomization studies are increasingly recognized as problematic,^52^ to address which, adherence to the STROBE-MR checklist for better appraisal may help.^22^ Only one of all included studies included STROBE-MR checklist. Having said that, we argued that careful design of the Mendelian randomization studies is perhaps more important, as conducting Mendelian randomization studies without critical thought on biological relevance may risk generating noises,^53^ or even misleading findings (e.g. instrumenting air pollution).^54^ Second, our analysis was limited to empirical evidence from COVID-19 related studies to reduce the risk of collider bias,^9^ which can be introduced via adjusting for the disease status when assessing the relation of instruments with putative outcomes. COVID-19 instruments may be atypical given changes in disease susceptibility throughout the pandemic arising from changes in public polices (e.g., mandatory vaccination) and human behaviors (e.g. mask wearing, social distancing) and may be argued as atypical. However, we also observed similar phenomenon in previous studies such as liability to type 2 diabetes in non-diabetic population,^14^ and association of liability to smoking in non-smoking populations.^55^

This review highlights the possible hurdles associated with using disease status as an exposure in Mendelian randomization by systematically reviewing COVID-19 Mendelian randomization studies. A better understanding of the concept of “relevance” in the context of 2 sample Mendelian randomization, and deliberation of study design with biological understanding, could greatly enhance the interpretability of findings from such studies that use binary traits as exposures, such as those exploring the impact of long COVID-19 using this design.

## Supplementary Material

Supplementary information

## Data Availability

The data from this systematic review were extracted from existing publications of Mendelian randomization studies.

## Textbox 1

Text box illustrating some examples with correct and incorrect interpretation of the findings from 11 Mendelian randomization studies reporting exposure as genetic liability of COVID-19

## References

[1] Davies NM, Holmes MV, Davey Smith G (2018). Reading Mendelian randomisation studies: a guide, glossary, and checklist for clinicians. Bmj.

[2] Morris AP, Voight BF, Teslovich TM, Ferreira T, Segre AV, Steinthorsdottir V (2012). Large-scale association analysis provides insights into the genetic architecture and pathophysiology of type 2 diabetes. Nat Genet.

[3] Suzuki K, Hatzikotoulas K, Southam L, Taylor HJ, Yin X, Lorenz KM (2024). Genetic drivers of heterogeneity in type 2 diabetes pathophysiology. Nature.

[4] Zeng P, Wang T, Zheng J, Zhou X (2019). Causal association of type 2 diabetes with amyotrophic lateral sclerosis: new evidence from Mendelian randomization using GWAS summary statistics. BMC Med.

[5] Pan Y, Wang Y, Wang Y (2020). Investigation of Causal Effect of Atrial Fibrillation on Alzheimer Disease: A Mendelian Randomization Study. J Am Heart Assoc.

[6] Budu-Aggrey A, Joyce S, Davies NM, Paternoster L, Munafo MR, Brown SJ (2021). Investigating the causal relationship between allergic disease and mental health. Clin Exp Allergy.

[7] De Silva NMG, Borges MC, Hingorani AD, Engmann J, Shah T, Zhang X (2019). Liver Function and Risk of Type 2 Diabetes: Bidirectional Mendelian Randomization Study. Diabetes.

[8] Au Yeung SL, Borges MC, Lawlor DA, Schooling CM (2022). Impact of lung function on cardiovascular diseases and cardiovascular risk factors: a two sample bidirectional Mendelian randomisation study. Thorax.

[9] Burgess S, Labrecque JA (2018). Mendelian randomization with a binary exposure variable: interpretation and presentation of causal estimates. Eur J Epidemiol.

[10] Leppert B, Riglin L, Wootton RE, Dardani C, Thapar A, Staley JR (2021). The Effect of Attention Deficit/Hyperactivity Disorder on Physical Health Outcomes: A 2-Sample Mendelian Randomization Study. Am J Epidemiol.

[11] Wu X, Zhang W, Zhao X, Zhang L, Xu M, Hao Y (2023). Investigating the relationship between depression and breast cancer: observational and genetic analyses. BMC Med.

[12] Holmes MV, Davey Smith G (2019). Can Mendelian Randomization Shift into Reverse Gear?. Clin Chem.

[13] Brenowitz WD, Zimmerman SC, Filshtein TJ, Yaffe K, Walter S, Hoffmann TJ (2021). Extension of Mendelian Randomization to Identify Earliest Manifestations of Alzheimer Disease: Association of Genetic Risk Score for Alzheimer Disease With Lower Body Mass Index by Age 50 Years. Am J Epidemiol.

[14] Bell JA, Bull CJ, Gunter MJ, Carslake D, Mahajan A, Davey Smith G (2020). Early Metabolic Features of Genetic Liability to Type 2 Diabetes: Cohort Study With Repeated Metabolomics Across Early Life. Diabetes Care.

[15] Smith ML, Bull CJ, Holmes MV, Davey Smith G, Sanderson E, Anderson EL (2023). Distinct metabolic features of genetic liability to type 2 diabetes and coronary artery disease: a reverse Mendelian randomization study. EBioMedicine.

[16] Au Yeung SL, Jiang C, Cheng KK, Liu B, Zhang W, Lam TH (2013). Is aldehyde dehydrogenase 2 a credible genetic instrument for alcohol use in Mendelian randomization analysis in Southern Chinese men?. Int J Epidemiol.

[17] Luykx JJ, Lin BD (2021). Are psychiatric disorders risk factors for COVID-19 susceptibility and severity? a two-sample, bidirectional, univariable, and multivariable Mendelian Randomization study. Transl Psychiatry.

[18] Liu D, Zhang Q, Bai P, Zhao J (2022). Assessing causal relationships between COVID-19 and non-alcoholic fatty liver disease. J Hepatol.

[19] Ran S, Pan L, Liu B (2022). Are patients with systemic lupus erythematosus at increased risk for COVID-19? A bi-directional Mendelian randomisation study. Lupus Sci Med.

[20] Liberati A, Altman DG, Tetzlaff J, Mulrow C, Gotzsche PC, Ioannidis JP (2009). The PRISMA statement for reporting systematic reviews and meta-analyses of studies that evaluate health care interventions: explanation and elaboration. PLoS Med.

[21] Moher D, Liberati A, Tetzlaff J, Altman DG (2009). Preferred reporting items for systematic reviews and meta-analyses: the PRISMA statement. PLoS Med.

[22] Skrivankova VW, Richmond RC, Woolf BAR, Yarmolinsky J, Davies NM, Swanson SA (2021). Strengthening the Reporting of Observational Studies in Epidemiology Using Mendelian Randomization: The STROBE-MR Statement. Jama.

[23] Luo S, Liang Y, Wong THT, Schooling CM, Au Yeung SL (2022). Identifying factors contributing to increased susceptibility to COVID-19 risk: a systematic review of Mendelian randomization studies. Int J Epidemiol.

[24] Niemi MEK, Karjalainen J, Liao RG, Neale BM, Daly M, Ganna A (2021). Mapping the human genetic architecture of COVID-19. Nature.

[25] Ellinghaus D, Degenhardt F, Bujanda L, Buti M, Albillos A, Invernizzi P (2020). Genomewide Association Study of Severe Covid-19 with Respiratory Failure. N Engl J Med.

[26] Pairo-Castineira E, Clohisey S, Klaric L, Bretherick AD, Rawlik K, Pasko D (2021). Genetic mechanisms of critical illness in COVID-19. Nature.

[27] Jia M, Chen HJ, Jia LM, Chen YL (2022). Genetic Predisposition to Coronavirus Disease 2019 in Relation to Ten Cardiovascular Conditions: A Two-Sample Mendelian Randomization Study. Front Med (Lausanne).

[28] Li J, Bai H, Qiao H, Yao P, Zhang Y (2023). Causal effects of COVID-19 on cancer risk: A Mendelian randomization study. J Med Virol.

[29] Gao R, Xu Y, Zhu G, Zhou S, Li H, Han G (2022). Genetic variation associated with COVID-19 is also associated with endometrial cancer. J Infect.

[30] Miao JP, Gu XY, Shi RZ (2022). COVID-19 is associated with the risk of cardiovascular disease death: A two-sample Mendelian randomization study. Front Cardiovasc Med.

[31] Baranova A, Cao H, Zhang F (2023). Causal effect of COVID-19 on Alzheimer’s disease: A Mendelian randomization study. J Med Virol.

[32] Tan JS, Liu NN, Guo TT, Hu S, Hua L (2021). Genetic predisposition to COVID-19 may increase the risk of hypertension disorders in pregnancy: A two-sample Mendelian randomization study. Pregnancy Hypertens.

[33] Gao X, Wei T, Wang H, Sui R, Liao J, Sun D (2023). Causal associations between obstructive sleep apnea and COVID-19: A bidirectional Mendelian randomization study. Sleep Med.

[34] Zhang Z, Fang T, Lv Y (2022). Causal associations between thyroid dysfunction and COVID-19 susceptibility and severity: A bidirectional Mendelian randomization study. Front Endocrinol (Lausanne).

[35] Au Yeung SL, Jiang CQ, Cheng KK, Liu B, Zhang WS, Lam TH (2012). Evaluation of moderate alcohol use and cognitive function among men using a Mendelian randomization design in the Guangzhou biobank cohort study. Am J Epidemiol.

[36] Rosenberg NA, Huang L, Jewett EM, Szpiech ZA, Jankovic I, Boehnke M (2010). Genome-wide association studies in diverse populations. Nat Rev Genet.

[37] Au Yeung SL, Zhao JV, Schooling CM (2021). Evaluation of glycemic traits in susceptibility to COVID-19 risk: a Mendelian randomization study. BMC Med.

[38] Leong A, Cole JB, Brenner LN, Meigs JB, Florez JC, Mercader JM (2021). Cardiometabolic risk factors for COVID-19 susceptibility and severity: A Mendelian randomization analysis. PLoS Med.

[39] Fernandez-Gallego N, Castillo-Gonzalez R, Mendez-Barbero N, Lopez-Sanz C, Obeso D, Villasenor A (2022). The impact of type 2 immunity and allergic diseases in atherosclerosis. Allergy.

[40] Heppner FL, Ransohoff RM, Becher B (2015). Immune attack: the role of inflammation in Alzheimer disease. Nat Rev Neurosci.

[41] Levey DF, Galimberti M, Deak JD, Wendt FR, Bhattacharya A, Koller D (2023). Multi-ancestry genome-wide association study of cannabis use disorder yields insight into disease biology and public health implications. Nat Genet.

[42] Fischer B, Lindner SR, Hall W (2022). Cannabis use and public health: time for a comprehensive harm-to-others framework. Lancet Public Health.

[43] UN Publication (2022). World Drug Report 2022.

[44] Chapman SJ, Hill AV (2012). Human genetic susceptibility to infectious disease. Nat Rev Genet.

[45] Stein CM (2011). Genetic epidemiology of tuberculosis susceptibility: impact of study design. PLoS Pathog.

[46] Bostock CV, Soiza RL, Whalley LJ (2009). Genetic determinants of ageing processes and diseases in later life. Maturitas.

[47] Cheung CL, Ho SC, Krishnamoorthy S, Li GH (2022). COVID-19 and platelet traits: A bidirectional Mendelian randomization study. J Med Virol.

[48] Li M, Yeung CHC, Schooling CM (2021). Circulating Cytokines and Coronavirus Disease: A BiDirectional Mendelian Randomization Study. Front Genet.

[49] Zhu G, Zhou S, Xu Y, Gao R, Li H, Su W (2022). Mendelian randomization study on the causal effects of COVID-19 on childhood intelligence. J Med Virol.

[50] Zhang K, Gao H, Chen M (2022). Genetic susceptibility to COVID-19 may increase the risk of erectile dysfunction: A two-sample Mendelian randomization study. Andrologia.

[51] Cao H, Baranova A, Wei X, Wang C, Zhang F (2023). Bidirectional causal associations between type 2 diabetes and COVID-19. J Med Virol.

[52] Kjaergaard AD, Smith GD, Stewart P (2024). Mendelian Randomization Studies in Endocrinology: Raising the Quality Bar for Submissions and Publications in The Journal of Clinical Endocrinology &amp; Metabolism. The Journal of Clinical Endocrinology & Metabolism.

[53] Au Yeung SL, Gill D (2023). Standardizing the reporting of Mendelian randomization studies. BMC Med.

[54] Au Yeung SL, Gill D (2024). Concerns over using the Mendelian randomization design to investigate the effect of air pollution. Sci Total Environ.

[55] Reed ZE, Wootton RE, Khouja JN, Richardson TG, Sanderson E, Davey Smith G (2025). Exploring pleiotropy in Mendelian randomisation analyses: What are genetic variants associated with ‘cigarette smoking initiation’ really capturing?. Genetic Epidemiology.

