# Supplementary material for: Challenges in interpreting Mendelian randomization studies with a disease as the exposure: Using COVID-19 liability studies as an exemplar
Source: Eur J Hum Genet. Author manuscript; Available in PMC 2025 Aug 14. (PMC12048694; doi:10.1038/s41431-025-01840-x)
Supplement: Supplementary information [file EMS207586-supplement-Supplementary_information.zip › 41431_2025_1840_MOESM2_ESM.docx]

PubMed (20 May 2023)

1. (covid-19 OR sars-cov-2 OR 2019-nCoV OR (novel coronavirus) OR (severe acute respiratory syndrome coronavirus 2) OR COVID) Filter: 2019/01/01-2023/04/13; language: English Species: humans
2. (1) AND (mendelian randomization) N=148
3. (1) AND (genome wide association study) N=296
4. (1) AND (mendelian randomisation) N=148
5. (1) AND (GWAS) N=312

Total: N=363

EMBASE (20 May 2023)

1. (covid-19 or sars-cov-2 or 2019-nCoV or COVID or severe acute respiratory syndrome coronavirus 2 or novel coronavirus).af. N= 435700
2. (mendelian randomization or genome wide association study or mendelian randomisation or GWAS).af.     N=74133
3. 1 and 2 N=821
4. limit 3 to (human and English.lg. and yr="2019 -Current")

Total: N=772

Ovid MEDLINE(R) (20 May 2023)

1. (covid-19 or sars-cov-2 or 2019-nCoV or COVID or severe acute respiratory syndrome coronavirus 2 or novel coronavirus).af. N=234121
2. (mendelian randomization or genome wide association study or mendelian randomisation or GWAS).af.     N=49327
3. 1 and 2 N=340
4. limit 3 to (human and English.lg. and yr="2019 -2021")

Total: N=339
